# 

**Published:** 1954-10

**Authors:** 


MEDICAL JOURNAL OF THE SOUTH-WEST

*(Incorporating the Bristol Medico-Chirurgical Journal)*

Established 1883

VOL. 70 (iv) October, 1954 NO. 256

### Contents

*PUBLISHED QUARTERLY*

*ANNUAL SUBSCRIPTION 16/- POST FREE*

PUBLISHED UNDER THE AUSPICES OF THE
BRISTOL MEDICO-CHIRURGICAL SOCIETY

### *Editorial Committee*

MR. H. KEITH LUCAS, 60 St. John's Road, Bristol 8. *Editor.*

DR. J. E. CATES, Dept. of Medicine, Bristol Royal Infirmary } *Assistant Editors.*
DR. A. H. GALE, The University, Bristol 8 }

DR. J. MACRAE, Ham Green Hospital. *Treasurer.*

MR. J. P. PARTRIDGE, Dept. of Surgery, Bristol Royal Infirmary.

*Local Correspondents*

*Bath* . . Mr. A. D. BATEMAN, 3 The Circus, Bath.
*Exeter* . . Dr. L. N. JACKSON, Mount Jocelyn, Crediton, Devon.
*Gloucester* . Dr. R. F. JARRETT, 9 College Green, Gloucester.
*Plymouth* . Dr. C. R. CROFT, Lexden, Hartley Av., Plymouth.
*Taunton* . Dr. M. MCALLEY, 1 The Crescent, Taunton.
*Torquay* . Dr. A. ROBINSON THOMAS, 20 Devon Square, Newton Abbot, Devon.
*Truro* . . Dr. F. D. M. HOCKING, St. Andrews, Perranporth, Cornwall.
*Yeovil* . . Mr. J. A. VERE NICOLL, Knapp House, Yeovil, Somerset.

### NOTICE TO CONTRIBUTORS

Original articles are invited, provided they have not been published elsewhere. Notes of forthcoming events, meetings held, degrees conferred, staff appointments, and other local news are welcomed. The editorial committee reserves the right to make literary corrections.

Illustrations should always be supplied ready for reproduction. All re-touchings or other operations required will be charged to the author. Line drawings should be drawn in Indian ink. We regret that we must ask authors to pay for making blocks, if this is necessary.

J. W. ARROWSMITH LTD., WINTERSTOKE ROAD,
BRISTOL.

### SPARE COPIES WANTED

*Medical Journal of the South-West*

Vol. 70 (i) 1953, No. 253

The editor would be glad to receive a number of copies of this issue to complete several exchanges for foreign journals.

If you can spare your copy of this issue, please send it to Mr. A. E. Roberts, Medical Librarian, University of Bristol, or leave it at the B.R.I. for the editor.

### Notes and News

Mr. R. V. Cooke has been appointed to a Hunterian Professorship of the Royal College of Surgeons. His subject will be "Advanced Carcinoma of the Colon".

Surgeon Commander J. L. S. Coulter, D.S.C., edited the Naval Medical Services volume of the Official Medical History of the War. He has been awarded the Gilbert Blane Medal.

The following have left or are leaving shortly to take up consultant posts elsewhere:
Dr. G. M. Colson, Senior registrar (medicine) to Bolton.
Dr. B. M. Laurence, Senior registrar (paediatrics) to Derby.
Mr. P. S. London, Senior registrar (orthopaedics) to Birmingham.
Mr. H. F. T. MacFetridge, Senior registrar (ophthalmology) to Herefordshire.
Mr. P. Ramsay, M.B.E., Senior registrar (surgery) to Windsor.
Mr. T. F. Redman, Lecturer (Obstetrics & Gynaecology) to Leeds.
Dr. I. M'D. G. Stewart, Senior registrar (medicine) to Blackpool.

*Birthday Honours:* R. F. Bolt, O.B.E.

*M.D. (Bristol):* N. C. Tanner.

*F.R.C.S. (England):* W. J. Gall.

*M.R.C.P. (London):* June K. Lloyd, C. D. R. Pengelly, R. H. R. White.

### UNIVERSITY OF BRISTOL

### *M.B., Ch.B. (BRISTOL) (JUNE,* 1954)

WITH FIRST-CLASS HONOURS

Mary E. Moore, (Distinction in Child Health, in Surgery, and in Obstetrics).

WITH SECOND-CLASS HONOURS

K. R. Gough (Distinction in Public Health and in Obstetrics); D. B. Paintin (Distinction in Obstetrics).

PASS

G. A. J. Ayliffe.

J. F. Baden-Daintree (Distinction in Public Health).

V. Baker.

C. A. Bickford.

J. M. B. Burn.

J. T. Bush.

T. H. P. Chatfield.

J. R. Clamp (Distinction in Public Health).

Mavis E. Coutts.

G. G. Crabbe.

Violet E. Dadds.

R. N. Davies.

G. N. Febry.

D. W. Fortune.

Angela C. Fowler.

D. B. Fowler.

J. S. Hughes-Games.

J. L. James (Distinction in Child Health).

F. E. S. Keiller.

Jean D. Kelly.

C. A. Milner.

E. J. R. Morgan.

D. G. Osmond.

J. M. V. Packer.

Ruth D. Penn.

M. Roylance.

R. K. M. Sanders.

H. Savery.

R. Seymour-Shove (Distinction in Public Health).

F. G. Spear.

D. M. M. Thompson.

D. H. Waldron.

M. R. Wills (Distinction in Public Health).

G. C. B. Winter.

### MEDICAL POSTGRADUATE DEPARTMENT PROGRAMME

October 24th, 10.30 a.m., Department of Medicine, Royal Infirmary: "**Cortisone and A.C.T.H.**" Dr. J. E. Cates.

October 31st, 10.30 a.m., X-ray Department, Southmead: "**Pulmonary Tuberculosis.**" Dr. A. T. M. Roberts.

November 7th, 10.30 a.m., X-ray Department, Southmead: "**Pulmonary Tuberculosis.**" Dr. A. T. M. Roberts.

November 13th } Course in Civil Defence arranged by Regional Hospital Board.

November 14th }

November 21st, at 10.30 a.m. and November 28th, at 10.30 a.m.: Dr. R. F. Barbour and the staff of the Child Guidance Clinic will give two demonstrations at the Child Guidance Clinic 7 Brunswick Square, Bristol 2.

### Appointments

### BRISTOL

| Name | Appointment | Formerly |
| --- | --- | --- |
| GORDON, IAN RONALD SIMSON, M.D., M.R.C.P., D.M.R.D. | Consultant Radiologist | Locum Tenens Assistant Radiologist, United Bristol Hospitals) S.H.M.O. Grade) |
| LAMB, W. R., B.A., M.B., B.CH. (DUBLIN), F.R.C.S.I. | Anaesthetic Registrar, Southmead Hospital, Bristol. | Senior House Officer in Anaesthetics, United Liverpool Hospitals. |
| TOVEY, L. A. D., M.B., CH.B. (BRISTOL). | Registrar to the South West Regional Blood Transfusion | Registrar in Pathology, County Laboratory, Dorchester. |
| FOX, J. H., M.B., CH.B. (SHEFFIELD), D.P.H. (EDIN.). | Registrar in Diseases of the Chest, Bristol Clinical Area. | Senior House Officer in Diseases of the Chest, Ham Green Hospital. |
| LAIDLAW, C. D'ARCY, M.R.C.S., L.R.C.P., F.R.C.S. (ENG.). | Surgical Registrar, Southmead Hospital, Bristol. | Surgical Registrar, St. Chad's Hospital, Birmingham. |
| DUTTON, J. E. M., M.B., B.S. (DURHAM), F.R.C.S. (ENG.). | Senior Registrar to the Dept. of Neurological Surgery, Frenchay Hospital, Bristol. | Registrar to the Dept. of Neurological Surgery, Frenchay Hospital. |
| LENNARD, M. B., M.B., CH.B. (BRISTOL), M.R.C.S., L.R.C.P. | Clinical Assistant in Orthopaedic & Traumatic Surgery, Weston-super-Mare General Hospital. | General practice. |
| CATTON, M. J., M.B., B.S. (LONDON). | Registrar in Pathology, Southmead Hospital, Bristol. | Graded Specialist in Pathology, R.A.F. |
| MILLS, C. P., M.B., B.S., F.R.C.S. | E.N.T. Registrar. | Surgical Registrar, Royal Hampshire County Hospital, Winchester. |
| CANTRELL, G. L. M.B., CH.B., D.O. | Senior Registrar in Ophthalmology. | Registrar in Ophthalmology, United Bristol Hospitals. |
| TULLOCH, L. G., M.B., CH.B., M.D. | Senior Registrar in Dermatology to Bristol Clinical Area based at U.B.H. (*R.H.B. appointment*) | Registrar, Manchester & Salford Hospital for Diseases of the Skin. |
| LLOYD, MISS J. K., M.B. CH.B. | Registrar in Paediatrics. | S.H.O. in General Medicine, United Bristol Hospitals. |
| BENNETT, J. R., B.A., M.B., B.CHIR., F.F.A.R.C.S. | Registrar in Anaesthetics. | Registrar in Anaesthetics, United Liverpool Hospitals. |
| FARRANT, P. C., M.B., CH.B., M.R.C.S., L.R.C.P. | Registrar in Pathology. | Demonstrator in Pathology, University of Bristol. |
| MORGAN, O. T. L., M.B., B.S., D.A. | Senior Registrar in Anaesthetics. | Registrar in Anaesthetics, United Bristol Hospitals. |
| ISAACSON, A. H., M.D., M.R.C.P. | Part-time Registrar in Radio-diagnosis in conjunction with University D.M.R.D. Course. | General Practice, Preston. |

### NORTH GLOUCESTERSHIRE

| Name | Appointment | Formerly |
| --- | --- | --- |
| RUDDELL, J. S., B.A., M.B., B.CH., B.A.O., D.A., M.A., M.D. | Consultant Anaesthetist, North Gloucestershire Clinical Area. | Consultant Anaesthetist, Northallerton Group of Hospitals. |

### *NORTH GLOUCESTERSHIRE*—continued.

| *Name* | *Appointment* | *Formerly* |
|---|---|---|
| BALMFORTH, G. V., M.R.C.S., L.R.C.P., M.B., B.S., D.(OBST.), R.C.O.G. | Medical Registrar, Gloucestershire Royal Hospital, Gloucester. | Trainee Assistant in General Practice in Barnstaple. |
| MORGAN, RHONA E., M.B., B.CH. (WALES), D.(OBST.)R.C.O.G., D.C.H. | Medical Registrar, Cheltenham General Hospital. | Senior House Officer (General Medicine), Stoke Mandeville Hospital. |
| CUSACK, J. J., M.B., B.CH. (DUBLIN) | Clinical Assistant in Ophthalmology, Cheltenham General Hospital (4 sessions). | Surgeon Commander, Royal Navy—Ophthalmic Specialist. |
| JATOI, A. M., M.B., B.S. (BOMBAY). | Orthopaedic Registrar, Gloucestershire Royal Hospital, Gloucester. | Senior House Officer in Orthopaedics, Gloucestershire Royal Hospital. |

### *BATH*

| *Name* | *Appointment* | *Formerly* |
|---|---|---|
| GERRARD, JEAN O., M.B., CH.B. (ST. ANDREWS') | Anaesthetic Registrar to the Bath Group of Hospitals. | Senior House Officer in Anaesthetics, St. Martin's Hospital, Bath. |
| POYNER-WALL, PHYLLIS, M.B., B.S. (LONDON), M.R.C.S., L.R.C.P., D.C.H. | Deputy Resident Physician, Winsley Chest Hospital, near Bath. | Registrar in Diseases of the Chest, Winsley Chest Hospital. |
| STEVENS, D. M., M.R.C.S., L.R.C.P., M.B., B.S., D.L.O. (ENG.), F.R.C.S. (EDIN.). | Consultant E.N.T. Surgeon to the Bath Clinical Area. | Senior Registrar in E.N.T. Surgery, United Cardiff Hospitals. |
| BAZELEY, R. W., M.R.C.S., L.R.C.P., M.B., B.S. | Re-appointed for a further year as Clinical Assistant in General Medicine, Royal United Hospital, Bath (1 session). | Is in general practice as Radstock. |
| PINCHING, J., M.R.C.S., L.R.C.P., B.M., B.CH., M.R.C.P. | Re-appointed for a further year as Clinical Assistant in General Medicine, Royal United Hospital, Bath (1 session). | Is in general practice at Wells. |
| IRWIN, T. W. N., M.B., B.CH. (WITWATERSRAND). | Surgical Registrar to the Bath Group of Hospitals. | Full-time study for the final F.R.C.S. (Eng.). |
| MATHESON, A. T., B.SC., M.B., CH.B. (CAPE TOWN), F.R.C.S. (EDIN.). | Surgical Registrar to the Bath Group of Hospitals. | Locum Surgical Registrar at the Central Middlesex Hospital. |
| RIVETT, P. A. H., M.B., CH.B. (LEEDS), M.D. (LEEDS), D.C.H. | Appointed for one year as Clinical Assistant in Paediatrics at the Royal United Hospital (1 session). | Is in general practice at Calne. |
| ARMIN, R. H., M.A., M.B., B.CHIR., M.R.C.S., L.R.C.P., D.P.M. | Consultant Psychiatrist and Deputy Physician Superintendent, Mendip Hospital, Wells. | Assistant Psychiatrist at Roundway Hospital, Devizes. |

### *SOUTH SOMERSET*

| *Name* | *Appointment* | *Formerly* |
|---|---|---|
| SINTON, J. R., M.B., B.S. (LOND.), M.R.C.P. (LOND.), M.D. (LOND.). | Consultant Chest Physician to the South Somerset Clinical Area. | Deputy Physician-Superintendent & Resident Assistant Physician, Baguley Hospital, Wythenshawe, Manchester. |

### *SOUTH SOMERSET*—continued.

| *Name* | *Appointment* | *Formerly* |
|---|---|---|
| HUGGILL, P. H., B.A., M.B., B.CH., M.R.C.S., L.R.C.P., D.L.O., F.R.C.S. (ENG.). | Consultant E.N.T. Surgeon to the South Somerset Clinical Area. | Part-time Consultant E.N.T. Surgeon to the Camberwell Group of Hospitals, part-time Senior Registrar to the E.N.T. Department at the Royal Cancer Hospital & Hon. Clinical Assistant to the E.N.T. Department at St. Mary's Hospital. |

### *DEVON AND CORNWALL*

| *Name* | *Appointment* | *Formerly* |
|---|---|---|
| DREW, D. W. A., L.D.S., R.C.S., M.R.C.S., L.R.C.P. | Anaesthetic Registrar to the Plymouth, South Devon & East Cornwall Hospital. | Anaesthetic Registrar at the United Cardiff Hospitals. |
| AZAM, M. A., M.B., B.S., (PUNJAB), F.R.C.S. (EDIN.). | Surgical Registrar at the Plymouth, South Devon & East Cornwall Hospital. | Senior House Officer at Shotley Bridge General Hospital. |
| PARKER, R. D. H., M.B., B.CH. (DUBLIN), F.R.C.S.I. | Surgical Registrar at the Plymouth, South Devon & East Cornwall Hospital. | Surgical Registrar at Worcester Royal Infirmary. |
| PASCALL, K. G., L.M.S.S.A., M.B., B.S., F.R.C.S. (EDIN.). | Senior Casualty Officer to the Plymouth, South Devon and East Cornwall Hospital Group. | Assistant Senior Administrative Medical Officer, H.Q. Staff of the South Western Regional Hospital Board. |
| PEARSON, A. F., M.B., CH.B. (MANCHESTER), D.(OBST.) R.C.O.G., M.R.C.O.G. | Clinical Assistant in Obstetrics & Gynaecology at Stratton Hospital, Bude, & Old Tree House Maternity Home, Launceston (1 session). | Is in general practice at Holsworthy. |
| WHEELDON, F. T., M.R.C.S., L.R.C.P., M.B., B.S. (LONDON), F.R.C.S. (ENG.). | Consultant Orthopaedic Surgeon, Plymouth Clinical Area. | Senior Registrar in Orthopaedic & Traumatic Surgery, St. George's Hospital, S.W.1. |
| GREENBURGH, H., M.R.C.S., L.R.C.P., M.B., B.S. (LOND.), M.R.C.P. (LOND.), M.D. (LOND.). | Consultant Pathologist, Plymouth Clinical Area. | Senior Registrar in Pathology, Southern Group Laboratory, Hither Green. |
| VARMA, R. M., M.B., B.S. (MADRAS). | Surgical Registrar at the Royal Cornwall Infirmary, Truro. | Senior House Officer to the Department of Neurological Surgery, Frenchay Hospital, Bristol. |
| RICH, W. M., M.R.C.S., L.R.C.P., D.O.M.S. | Clinical Assistant in Ophthalmology to the West Cornwall Clinical Area. | |
| BENNETT, H. S., M.B., CH.B. (ABERDEEN), D.M.R.D. (ENG.). | Consultant Radiologist to the West Cornwall Clinical Area. | Assistant Radiologist to the Department of Radiodiagnosis, Aberdeen General Hospitals. |

